# Amino acid substitutions in NSP6 and NSP13 of SARS-CoV-2 contribute to superior virus growth at low temperatures

**DOI:** 10.1128/jvi.02217-24

**Published:** 2025-02-12

**Authors:** Yuri Furusawa, Maki Kiso, Ryuta Uraki, Yuko Sakai-Tagawa, Hiroyuki Nagai, Michiko Koga, Yukie Kashima, Masayuki Hojo, Noriko Iwamoto, Kiyoko Iwatsuki-Horimoto, Norio Ohmagari, Yutaka Suzuki, Hiroshi Yotsuyanagi, Peter J. Halfmann, Wataru Kamitani, Seiya Yamayoshi, Yoshihiro Kawaoka

**Affiliations:** 1 The Research Center for Global Viral Diseases, National Center for Global Health and Medicine Research Institute 350198 , Shinjuku, Tokyo, Japan; 2 Division of Virology, Institute of Medical Science, University of Tokyo, Shinjuku, Tokyo, Japan; 3 The University of Tokyo Pandemic Preparedness, Infection and Advanced Research Center, Shinjuku, Tokyo, Japan; 4 Department of Infectious Diseases and Applied Immunology, IMSUT Hospital of Institute of Medical Science, The University of Tokyo, Shinjuku, Tokyo, Japan; 5 Division of Infectious Diseases, Advanced Clinical Research Center, Institute of Medical Science, The University of Tokyo, Shinjuku, Tokyo, Japan; 6 Department of Computational Biology and Medical Sciences, Graduate School of Frontier Sciences, The University of Tokyo, Shinjuku, Tokyo, Japan; 7 Department of Respiratory Disease, National Center for Global Health and Medicine, Shinjuku, Tokyo, Japan; 8 Disease Control and Prevention Center, National Center for Global Health and Medicine, Shinjuku, Tokyo, Japan; 9 Department of Pathobiological Sciences, School of Veterinary Medicine, University of Wisconsin-Madison 5228 , Madison, Wisconsin, USA; 10 Department of Infectious Diseases and Host Defense, Graduate School of Medicine, Gunma University, Gunma, Japan; 11 International Research Center for Infectious Diseases, Institute of Medical Science, University of Tokyo, Shinjuku, Tokyo, Japan; St. Jude Children's Research Hospital, Memphis, Tennessee, USA

**Keywords:** SARS-CoV-2, COVID-19, coronavirus

## Abstract

In general, severe acute respiratory syndrome coronavirus 2 (SARS-CoV-2) replicates well at 37°C, which is the temperature of the human lower respiratory tract, but it poorly at 30°C‒32°C, which is the temperature of the human upper respiratory tract. The replication efficiency of SARS-CoV-2 in the upper respiratory tract may directly affect its transmissibility. In this study, an XBB.1.5 isolate showed superior replicative ability at 32°C and 30°C, whereas most other Omicron sub-variant isolates showed limited growth. Deep sequencing analysis demonstrated that the frequencies of viruses possessing the NSP6-S163P and NSP13-P238S substitutions increased to more than 97% during propagation of the XBB.1.5 isolate at 32°C but did not reach 55% at 37°C. Reverse genetics revealed that these substitutions contributed to superior virus growth *in vitro* at these low temperatures by improving virus genome replication. Mutant virus possessing both substitutions showed slightly higher virus titers in the upper respiratory tract of hamsters compared to the parental virus; however, transmissibility between hamsters was similar for the mutant and parental viruses. Taken together, our findings indicate that NSP6-S163P and NSP13-P238S contribute to superior virus growth at low temperatures *in vitro* and in the upper respiratory tract of hamsters.

**IMPORTANCE:**

Severe acute respiratory syndrome coronavirus 2 (SARS-CoV-2) replicates efficiently at 37°C. However, the temperature of the human upper airway is 30°C–32°C. Therefore, the replicative ability of SARS-CoV-2 at low temperatures could influence virus replication in the upper airway and transmissibility. In this study, we assessed the growth of Omicron sub-variants at low temperatures and found that an XBB.1.5 isolate showed increased replicative ability. By deep sequencing analysis and reverse genetics, we found that amino acid changes in NSP6 and NSP13 contribute to the low-temperature growth; these changes improved RNA polymerase activity at low temperatures and enhanced virus replication in the upper airway of hamsters. Although these substitutions alone did not drastically affect virus transmissibility, in combination with other substitutions, they could affect virus replication in humans. Furthermore, since these substitutions enhance virus replication in cultured cells, they could be used to improve the production of inactivated or live attenuated vaccine virus.

## INTRODUCTION

Severe acute respiratory syndrome coronavirus 2 (SARS-CoV-2) was identified as the causative agent of COVID-19 at the end of 2019 (1, 2). Omicron variants emerged at the end of 2021 and are still circulating throughout the world. Although many Omicron sub-variants have been detected, the differences in their characteristics and the biological significance of the various amino acid substitutions in the non-spike proteins have not been fully explored.

Under cell culture conditions, SARS-CoV-2 replicates well at 37°C, which is the temperature of the human lower respiratory tract, whereas it generally shows reduced replication at 30°C‒32°C, which is the temperature of the human upper respiratory tract. Therefore, improved virus replicative ability at low temperatures could increase their propagation in the upper respiratory tract. Furthermore, high virus titers in the upper respiratory tract may increase the virus load in droplets and aerosols produced by an infected patient (3), leading to high transmissibility between humans (4–6). Therefore, improved virus replicative ability at low temperatures may result in higher transmissibility. Although the Omicron sub-variants BA.5 and BQ.1.1 do not replicate well at elevated temperatures, unlike Delta variant (7), the replicative ability of Omicron sub-variants at low temperatures has not been investigated until now.

In hamster models, ancestral isolates of SARS-CoV-2 efficiently transmit between hamsters (8), and similar transmissibility was observed for the B.1.1.7 (Alpha) and B.1.617 (Delta) variants (9–11). In contrast, airborne transmission was not observed for Omicron variants such as BA.1 and BA.2 (12, 13), and inefficient airborne transmission was observed for XBB.1.5 (11) and EG.5.1 (14). The underlying molecular mechanisms that cause the difference in transmissibility among Omicron sub-variants have remained unclear. Furthermore, the link between virus replicative ability at low temperatures and transmissibility has not been investigated.

In this study, we compared the replicative ability of various Omicron sub-variants at low temperatures and attempted to identify amino acid substitutions that were important for superior virus growth at low temperatures. We also investigated whether replicative ability at low temperatures in cultured cells correlated with virus replication and transmissibility in hamsters.

## RESULTS

### Virus growth of omicron sub-variants at low temperatures

**Fig 1:**
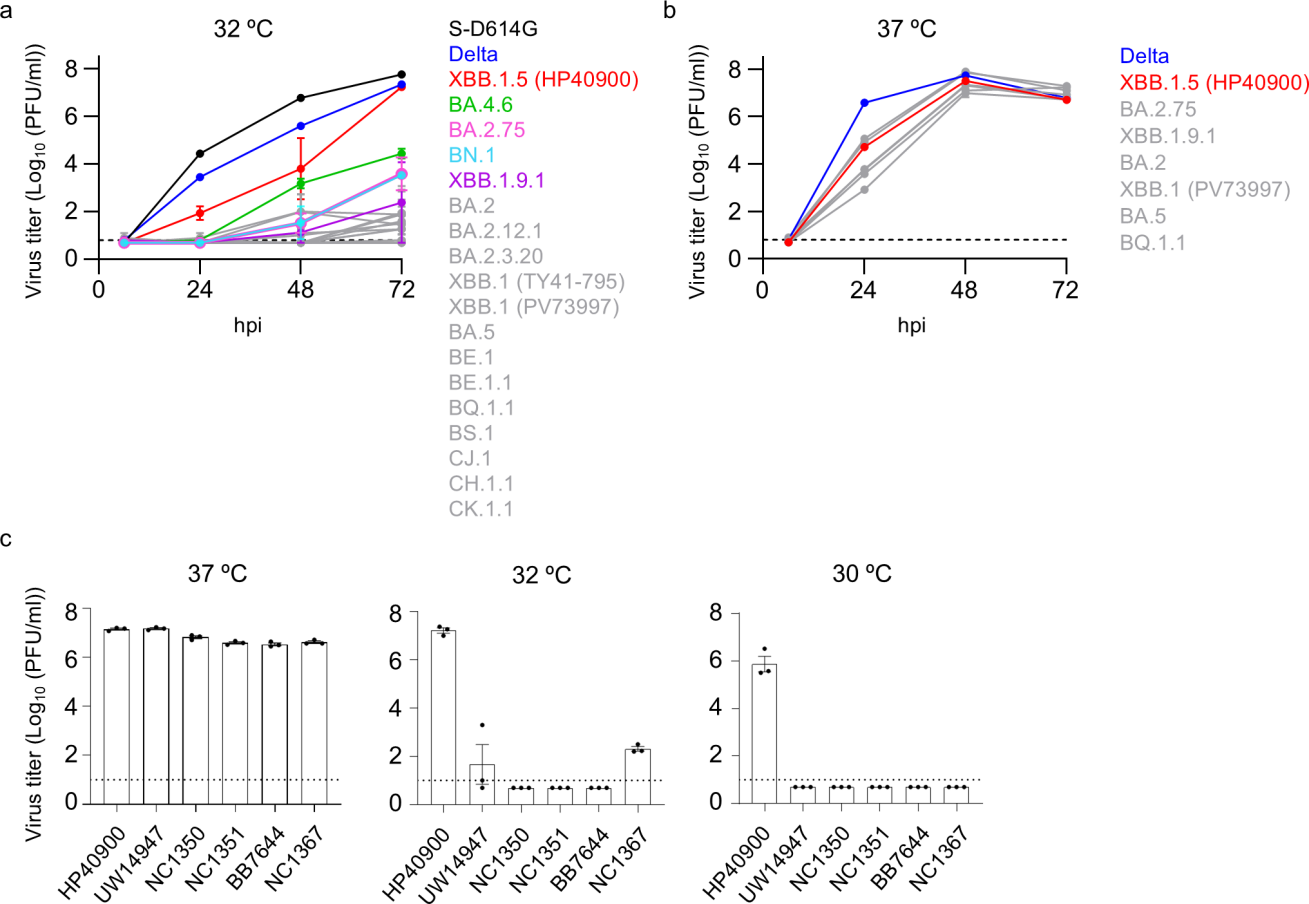
Growth kinetics of SARS-CoV-2 Omicron sub-variants at low temperatures. (a and b) VeroE6/TMPRSS2 cells at 32°C or 37°C were infected with the indicated virus at a multiplicity of infection (MOI) of 0.001. Virus titers at the indicated timepoints were determined by using plaque assays (*n* = 3, mean ± SEM). (c) VeroE6/TMPRSS2 cells were infected with six isolates of XBB.1.5 at an MOI of 0.001. The infected cells were incubated at 37°C, 32°C, or 30°C. Virus titers at 72 hpi were determined by using plaque assays (*n* = 3, mean ± SEM).

We assessed the growth of 18 Omicron sub-variants, as well as the S-D614G and Delta (B.1.617.2) variants, in VeroE6/TMPRSS2 cells at 32°C. While S-D614G and Delta replicated well at 32°C, most of the Omicron sub-variants showed limited replication (Fig. 1a, Table S1). However, one XBB.1.5 isolate, HP40900, showed relatively greater growth at 32°C (Fig. 1a). The replication of the Delta and Omicron sub-variants, including XBB.1.5 (HP40900), BA.2.75, XBB.1.9.1, BA.2, XBB.1 (PV73997), BA.5, and BQ.1.1, was also examined in VeroE6/TMPRSS2 cells at 37°C (Fig. 1b, Table S2). Delta showed a higher titer than the Omicron sub-variants at 24 hpi, but by 48 hpi, all of the Omicron sub-variants reached almost the same titer as Delta. The replicative ability of XBB.1.5 (HP40900) at 37°C was similar to that of the other Omicron sub-variants, suggesting that XBB.1.5 (HP40900) has superior growth only at the low temperature.

To confirm whether the superior replicative ability of XBB.1.5 (HP40900) at the low temperature was consistently observed among XBB.1.5 sub-variant isolates, we compared the growth of six XBB.1.5 sub-variant isolates (HP40900, UW14947, NC1350, NC1351, BB7644, and NC1367) at 37°C, 32°C, and 30°C. Although all six isolates grew well at 37°C, only HP40900 showed superior growth at 32°C and 30°C in VeroE6/TMPRSS2 cells (Fig. 1c), suggesting that some amino acid substitutions specific to HP40900 contribute to its superior growth at low temperatures.

### Amino acid substitutions in NSP6 and NSP13 are involved in superior growth at low temperatures

**Fig 2:**
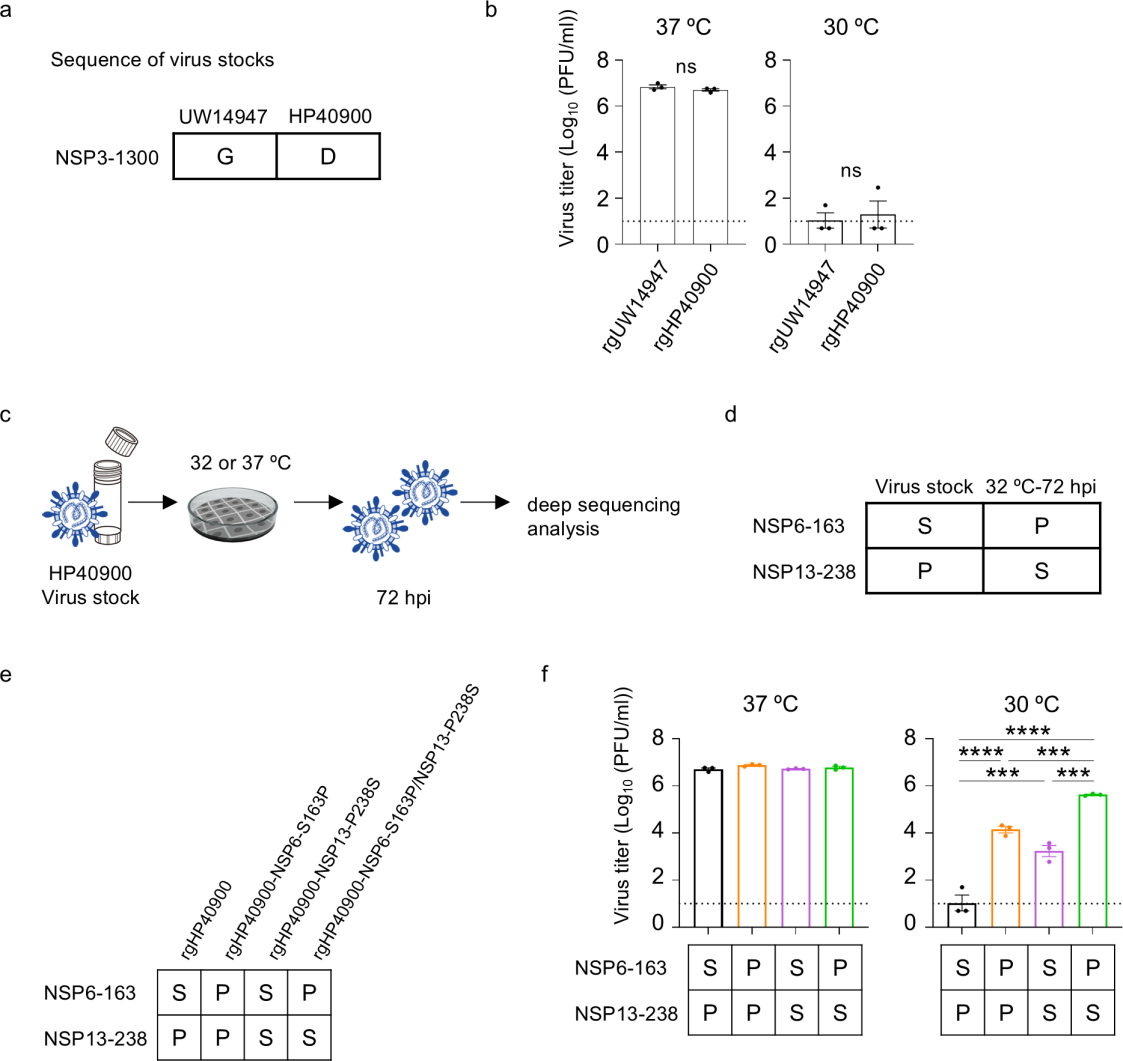
Identification of amino acid substitutions important for the low-temperature growth. (a) Amino acid difference between UW14947 and HP40900. (b) VeroE6/TMPRSS2 cells were infected with viruses generated by reverse genetics at a multiplicity of infection (MOI) of 0.001. The infected cells were incubated at 37°C or 30°C. Virus titers at 72 hpi were determined by using plaque assays (*n* = 3, mean ± SEM). (c) Schematic image of sample collection and deep sequencing analysis. (d) Results of deep sequencing analysis of the passaged virus. (e) Amino acid substitutions in generated viruses. (f) VeroE6/TMPRSS2 cells were infected with recombinant viruses at an MOI of 0.001. The infected cells were incubated at 37°C or 30°C. Virus titers at 72 hpi were determined by using plaque assays (*n* = 3, mean ± SEM). Data were analyzed by using a *t*-test (b) or one-way ANOVA with Dunnett’s multiple comparisons test (e). ∗*P* < 0.05, ∗∗*P* < 0.01, ∗∗∗*P* < 0.001, and ∗∗∗∗*P* < 0.0001; ns, not significant.

To identify amino acid substitutions that are important for superior growth at low temperatures, the amino acid sequences of the six XBB.1.5 isolates used in Fig. 1c were compared (Table S3). HP40900 was found to possess one strain-specific amino acid substitution: NSP3-G1300D (ORF1ab-G2118D). Therefore, we hypothesized that NSP3-G1300D was required for superior viral growth at low temperatures. To test this hypothesis, we generated rgHP40900 (NSP3-1300D) and rgUW14947 (NSP3-1300G) by reverse genetics using the bacterial artificial chromosome (BAC) system, since the amino acid sequence of these two isolates differed only at NSP3-1300 (Fig. 2a). The replicative ability of the rescued viruses in VeroE6/TMPRSS2 cells was examined at 37°C and 30°C. However, rgHP40900 (NSP3-1300D) showed a similar virus titer to rgUW14947 (NSP3-1300G) at both 37°C and 30°C (Fig. 2b), suggesting that NSP3-G1300D does not affect growth at low temperatures.

We then analyzed the genome sequences of HP40900 after a single passage in VeroE6/TMPRSS2 cells at 32°C or 37°C. Virus passage was performed in triplicate, and we found that the frequencies of some amino acid substitutions increased after passaging (Table S4). Among them, the frequencies of NSP6-S163P (ORF1ab-S3732P) and NSP13-P238S (ORF1ab-P5562S) increased to more than 90% in all three samples passaged at 32°C (Fig. 2d), whereas their frequencies did not reach 55% after passaging at 37°C (Table S4). This result suggests that viruses with these substitutions were selected under the low-temperature condition.

To elucidate the role of these two substitutions in low-temperature growth, we generated rgHP40900 possessing one or both of the substitutions (Fig. 2e). We then assessed the replicative ability of the mutant viruses at 37°C and 30°C in VeroE6/TMPRSS2 cells. The NSP6-S163P or NSP13-P238S substitution contributed to superior virus growth at 30°C and the combination of both substitutions further enhanced the replicative ability (Fig. 2f). Neither substitution affected virus growth at 37°C. Taken together, these results indicate that the NSP6-S163P and NSP13-P238S substitutions play a central role in the superior virus growth of HP40900 at low temperatures in VeroE6/TMPRSS2 cells.

### The roles of the NSP6 and NSP13 substitutions in superior growth at low temperatures

**Fig 3:**
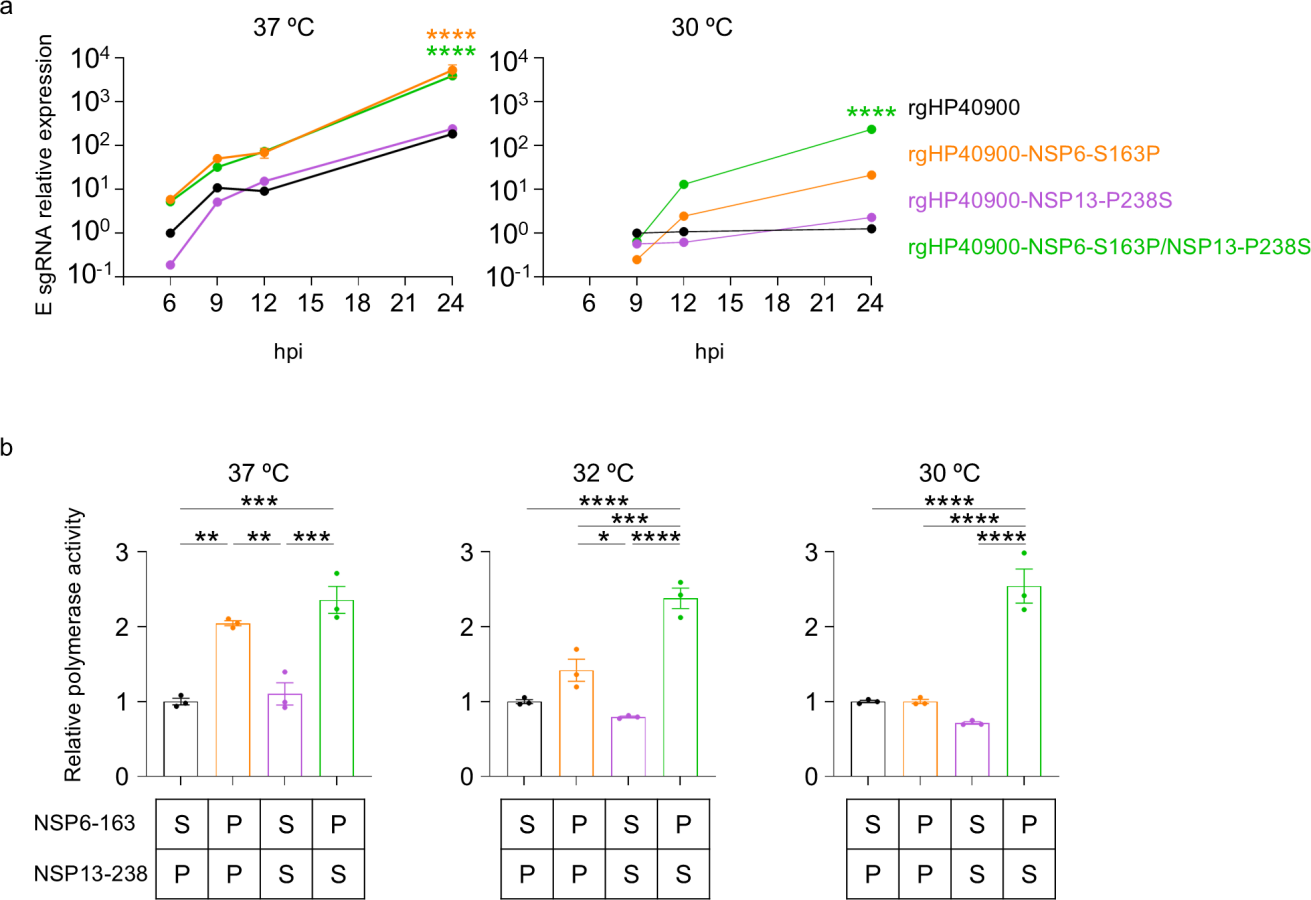
The role of NSP6-S163P and NSP13-P238S in virus transcription/replication. (a) VeroE6/TMPRSS2 cells were infected with each virus at a multiplicity of infection (MOI) of 0.5. At the indicated timepoints, total RNA was extracted from the infected cells, and the relative expression of sgRNA for E protein was measured by quantitative RT-PCR (*n* = 3, mean ± SEM). (b) Polymerase activity in HEK293T cells transfected with the replicative cDNA containing the nanoluciferase gene was determined at 72 h post-transfection (*n* = 3, mean ± SEM). Data were analyzed by using a two- (a) or one-way (b) ANOVA with Dunnett’s multiple comparisons test. ∗*P* < 0.05, ∗∗*P* < 0.01, ∗∗∗*P* < 0.001, and ∗∗∗∗*P* < 0.0001.

To reveal the roles of the NSP6-S163P and NSP13-P238S substitutions in superior virus growth at low temperatures, we examined virus genome replication in cells. For this, subgenomic RNAs (sgRNAs) are widely used because the expression level of sgRNAs is an indicator of active genome transcription and replication. Therefore, we quantified the sgRNA for the E protein in VeroE6/TMPRSS2 cells infected with rgHP40900, rgHP40900-NSP6-S163P, rgHP40900-NSP13-P238S, or rgHP40900-NSP6-S163P/NSP13-P238S at 37 and 30°C (Fig. 3a). We found that the NSP6-S163P substitution upregulated E sgRNA expression at both temperatures. Although the NSP13-P238S substitution alone did not alter E sgRNA expression at either 37°C or 30°C, it increased E sgRNA expression in combination with the NSP6-S163P substitution at 30°C. These results indicate that NSP6-S163P is involved in superior virus transcription/replication regardless of temperature, and NSP13-P238S plays a role in cold adaptation.

To further examine the roles of these substitutions in virus genome RNA replication, we used a DNA replicon system in which SARS-CoV-2 RNA containing the nanoluciferase gene is efficiently replicated by virus replicase, but infectious particles are not produced (15). We constructed replicon DNAs of HP40900 and its mutants and determined the expression level of nanoluciferase (i.e., virus polymerase activity) in cells transfected with either of the replicon DNAs. The NSP6-S163P substitution enhanced the polymerase activity significantly at 37°C and slightly at 32°C (Fig. 3b). Although the NSP13-P238S substitution alone did not affect polymerase activity, the combination of NSP6-S163P and NSP13-P238S significantly increased polymerase activity at 32°C and 30°C. These results suggest that the NSP6-S163P substitution enhances virus polymerase activity regardless of temperature and that the combination with NSP13-P238S is required for superior polymerase activity at low temperatures. Although NSP6-S163P alone increased sgRNA expression at 30°C, it did not increase polymerase activity in the DNA replicon system at 30°C. This inconsistency might be due to the differences between the assays and cells.

### The NSP6-S163P and NSP13-P238S substitutions increase virus replication in the upper respiratory tract of hamsters but not transmissibility

**Fig 4:**
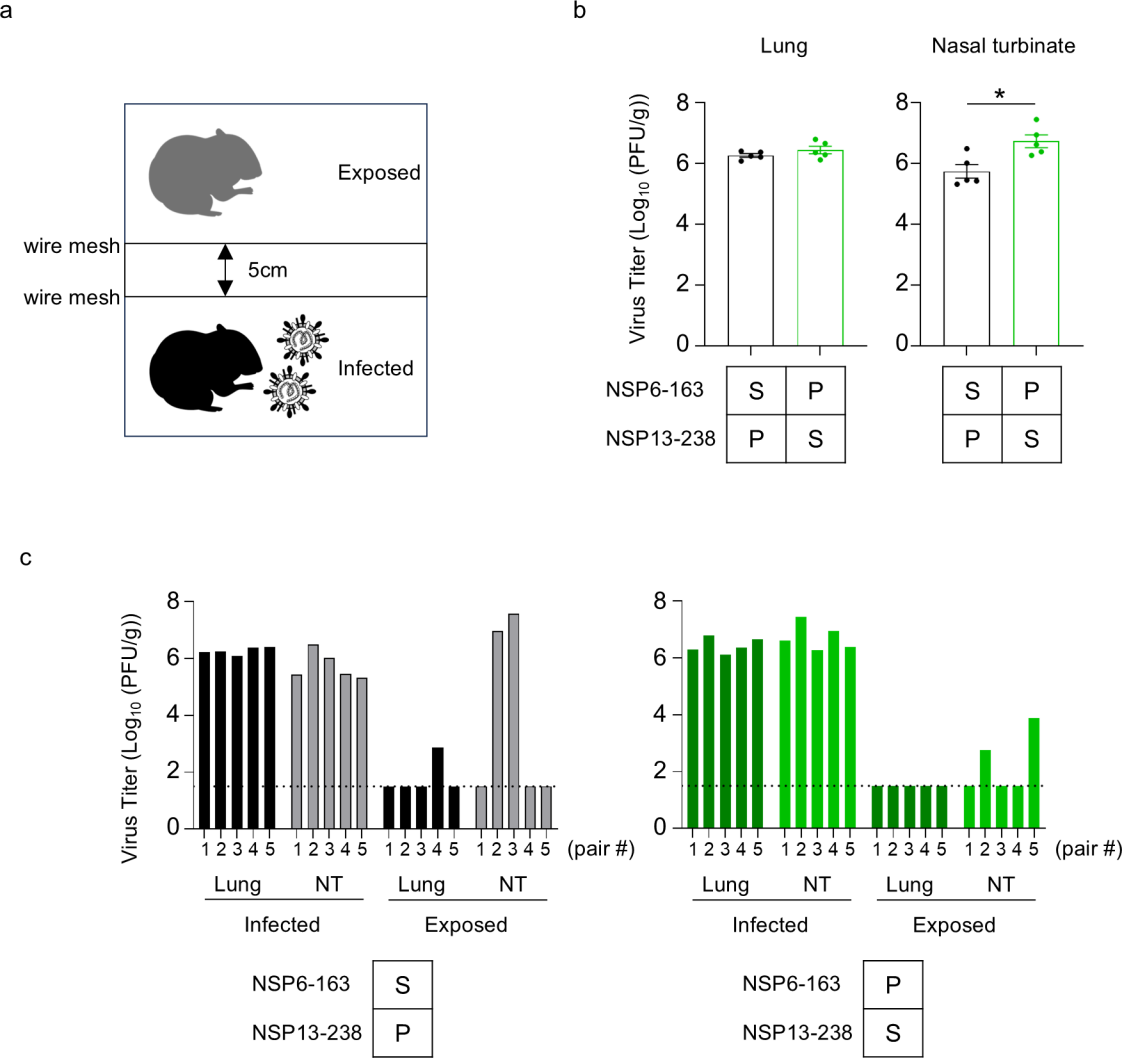
Replication and transmission of recombinant viruses in Syrian hamsters. (a) Schematic image of the *in vivo* experiment. (b and c) Syrian hamsters were intranasally inoculated with 10^4^ PFU (in 30 µL) of the indicated virus. Hamsters (*n* = 5) were euthanized at 4 days post-infection and 3 days post-exposure for virus titration. Virus titers in the nasal turbinates and lungs were determined by using plaque assays. (b) Vertical bars show the mean ± SEM. Points indicate data from individual hamsters. Data were analyzed by using a *t*-test. ∗*P* < 0.05. (c) The virus titers in the nasal turbinates and lungs of infected and exposed hamsters were determined by use of plaque assays on VeroE6/TMPRSS2 cells. Bars indicate the virus titer of each animal.

Since the NSP6-S163P and NSP13-P238S substitutions contributed to superior virus growth at low temperatures *in vitro*, we next examined their effect on replication in the respiratory tract and airborne transmissibility using Syrian hamsters, a well-established animal model for the study of SARS-CoV-2 (16). Hamsters were intranasally inoculated with rgHP40900 or rgHP40900-NSP6-S163P/NSP13-P238S, and the following day, naïve hamsters were exposed to the infected hamsters (Fig. 4a). The animals were physically separated by double-layered wire mesh to avoid direct contact but allow virus airborne transmission. After 72 h of exposure, virus titers in the nasal turbinates and lungs of the infected and exposed hamsters were measured. rgHP40900-NSP6-S163P/NSP13-P238S replicated better than rgHP40900 in the nasal turbinates, whereas both viruses replicated similarly in the lungs (Fig. 4b). In the exposed hamsters, infectious rgHP40900 was detected in the lungs of one of five hamsters and in the nasal turbinates of two of five hamsters. Infectious rgHP40900-NSP6-S163P/NSP13-P238S was detected in the nasal turbinates of two of five exposed hamsters (Fig. 4c). These results indicate that the NSP6-S163P and NSP13-P238S substitutions increase virus replication in the upper respiratory tract but may not affect transmissibility in the hamster model, although further analysis is needed to conclusively determine their contribution to transmissibility between humans.

## DISCUSSION

Since the emergence of Omicron at the end of 2021, numerous sub-variants with different amino acid substitutions in their non-structural and structural proteins have emerged. Because these amino acid substitutions have mainly accumulated in the spike protein, the differences they have brought about in antigenicity and receptor-binding capacity have been widely assessed. However, it has been unclear whether amino acid substitutions in the non-structural proteins confer differences in replicative ability. In this study, we investigated the replicative ability of various Omicron sub-variants at low temperatures and found that although Omicron sub-variants generally had low replicative ability at low temperatures, unlike the S-D614G and Delta variants, one isolate (HP40900) of the XBB.1.5-lineage showed relatively high replication at low temperatures. Deep sequencing analysis and reverse genetics revealed that the NSP6-S163P substitution contributes to the superior virus growth of this isolate by upregulating virus genome transcription and/or replication, and the NSP13-P238S substitution in combination with NSP6-S163P plays a central role in cold adaptation.

NSP6 is a transmembrane protein that plays a role in forming replication organelles (17, 18). Coronaviruses, including SARS-CoV-2, form a membrane-bound replication organelle composed of double-membrane vesicles (DMVs) and connectors in infected cells. DMVs are generated by NSP3 and NSP4 (19, 20). NSP6 is essential for the organization of DMVs and the connection between DMVs and the endoplasmic reticulum (ER) (17). Therefore, the NSP6-S163P substitution might influence replication organelle organization and enhance viral RNA replication. The structure of NSP6 has not been determined, and the predicted structure is controversial (17, 21–23), but NSP6-163 is predicted to be located near the border between the transmembrane and extracellular regions (24, 25). Truncation of the C-terminal region of NSP6, including NSP6-163S, resulted in the diffuse distribution of NSP6 in the ER (17). Therefore, the C-terminal region is required to generate the NSP6 compartment, which is necessary for its role in organizing DMVs, but it is not known whether NSP6-163S is involved in this function. Further analysis is required to determine the exact roles of the NSP6-S163P substitution. NSP6 has been reported to alter virus pathogenicity and contribute to the attenuation of Omicron BA.1 compared to Delta (26, 27). However, NSP6-163S is conserved between Delta and Omicron, suggesting that other amino acid substitutions in NSP6 are responsible for the attenuation of Omicron. In addition, we searched for NSP6 mutations in major Omicron subvariants and identified six mutations: NSP6-V24F, I189V, R252K, L260F, 105–107LSG deletion, and 106–108SGF deletion. The NSP6 of BA.1 bearing the 105–107LSG deletion and I189V attenuated viral replication (26, 27). The 105–107LSG deletion, which emerged in BA.1, and the 106–108SGF deletion, which emerged in BA.2, are reported to enhance type-I interferon antagonism (23). R252K and L260F are thought to enhance virus replication (28). Although it has been suggested that V24F does not significantly alter viral replication, its exact effect on viral characteristics remains unclear (28).

NSP13 belongs to the helicase superfamily 1B and catalyzes the unwinding of double-stranded DNA or RNA (29). It is the most conserved non-structural protein within the coronavirus family. SARS-CoV-2 NSP13 shows 99.8% identity to SARS-CoV-1 NSP13 (30). NSP13 interacts with NSP12 (viral RNA-dependent RNA polymerase) and functions as part of the replication-transcription complex together with NSP7, NSP8, and NSP12 (31). NSP13 has five domains: zinc-binding domain, stalk, 1B, 1A, and 2A (32). NSP13-238P is located in the 1B domain, suggesting that it might be involved in the interaction between the stalk domain and other domains that regulate unwinding activity (32). Therefore, the NSP13-P238S substitution might affect replication efficiency by improving unwinding activity. How NSP13 affects viral growth in combination with the NSP6 substitution specifically at low temperatures remains unclear, and further analysis is needed. When we searched for NSP13 mutations in major Omicron subvariants, we saw that NSP13-S36P emerged in XBB.1. However, no experimental studies have been performed on the functional significance of this specific mutation.

The NSP6-S163P and NSP13-P238S substitutions were associated with superior virus growth at low temperatures. Roles for NSP6 and NSP13 in cold adaptation have not previously been reported. Several cold-adapted SARS-CoV-2 strains that can replicate at temperatures lower than 37°C have been established, and amino acid substitutions detected after cold-adaptation have been reported (33–35). Although none of these cold-adapted viruses contained the NSP6-S163P or NSP13-P238S substitution, they harbored 31 amino acid substitutions including NSP6-F37L, NSP6-N40K, NSP13-L280F, and NSP13-V525I in one study (33), 20 amino acid substitutions including NSP6-I124T in a second study (34), and 23 amino acid substitutions including NSP13-D59N in a third report (35). Thus, although the substitutions that are responsible for cold adaptation have not yet been clearly identified, NSP6 and/or NSP13 of SARS-CoV-2 is likely hotspots for cold-adaptive mutation.

We also investigated virus replication and transmission in hamsters. For a virus possessing the NSP6-S163P and NSP13-P238S substitutions, the titers in the upper respiratory tract were slightly higher than that of the parental virus. Thus, the virus replicative ability at low temperatures *in vitro* is correlated with efficient virus replication in the upper respiratory tract. Although the NSP6-S163P and NSP13-P238S substitutions enhanced viral replication at low temperatures and in the nasal turbinate of hamsters, they did not enhance the airborne transmissibility of the virus. Therefore, their contributions to airborne transmissibility remain unknown.

When we performed virus passage in triplicate at 32°C or 37°C, the NSP6-S163P and NSP13-P238S substitutions were detected in all wells infected with HP40900 (Table S4). The frequency of viruses possessing these two substitutions increased to more than 97% at 32°C, whereas it increased to around 30%–50% at 37°C. By generating mutant viruses using reverse genetics, we demonstrated that the NSP6-S163P and NSP13-P238S substitutions enhanced virus growth *in vitro* at 30°C, which explains why the variant possessing these substitutions became predominant after passaging at 32°C. At 37°C, although viruses with or without these two substitutions replicated to similar titers, NSP6-S163P alone or in combination with NSP13-P238S enhanced the viral polymerase activity in replicon assays. These results may explain why viruses possessing these two substitutions became dominant but did not completely overtake the wild-type virus at 37°C. However, the reason why these substitutions occurred in HP40900 is unknown. We searched for these substitutions in clinical isolates submitted to the GISAID database and found that the prevalence of these substitutions is remarkably low, less than 0.05%, respectively. Therefore, viruses with these substitutions are not currently predominant in nature. The role of these two substitutions in humans is unknown. However, since our results suggest that these substitutions promote viral replication in the upper respiratory tract, the emergence of new variants with these substitutions should be carefully monitored.

In conclusion, we found that NSP6-S163P and NSP13-P238S detected in an isolate of XBB.1.5 improved viral RNA replication at low temperatures. These substitutions slightly increased virus replication in the upper respiratory tract but did not enhance transmissibility in hamsters. Although these substitutions alone did not drastically affect virus transmissibility, they increased virus RNA replication, especially at low temperatures. Therefore, it is possible that these two substitutions in combination with other substitutions might affect virus replication in humans. It has been reported that XBB.1.16 leads to a significantly higher hospitalization rate than other Omicron variants (36). XBB.1.16 has five amino acid substitutions compared to XBB.1.5: NSP6-L260F, NSP14-D222Y, S-E180V, S-T478R, and ORF9b-N55S. Therefore, the combination of NSP6-S163P and/or NSP13-P238S with these substitutions might affect viral replication and pathogenicity in humans. Furthermore, since these substitutions contribute to superior virus replication in cultured cells, they could be used to improve the production of inactivated or live attenuated vaccine virus.

## MATERIALS AND METHODS

### Cells

VeroE6/TMPRSS2 (JCRB 1819) cells (37, 38) were propagated in the presence of 1 mg/mL geneticin (G418; Invivogen) and 5 µg/mL plasmocin prophylactic (Invivogen) in Dulbecco’s modified Eagle’s medium (DMEM) containing 10% fetal bovine serum (FBS). Vero E6-TMPRSS2-T2A-ACE2 cells (provided by Dr. Barney Graham, NIAID Vaccine Research Center) were cultured in DMEM supplemented with 10% FBS, 10 mM HEPES pH 7.3, 100 U/mL penicillin–streptomycin, and 10 µg/mL puromycin. HEK293T cells were cultured in DMEM supplemented with 10% FBS. All cells were maintained at 37°C with 5% CO_2_. The cells were regularly tested and confirmed to be negative for mycoplasma contamination by using PCR.

### Viruses

The following SARS-CoV-2 viruses were isolated using VeroE6/TMPRSS2 cells or Vero E6-TMPRSS2-T2A-ACE2 cells and propagated in VeroE6/TMPRSS2 cells at 37°C: SARS-CoV-2/UT-HP095-1N/Human/2020/Tokyo (S-D614G), hCoV-19/USA/WI-UW-5250/2021 (Delta), hCoV-19/USA/MD-HP40900-PIDYSWHNUB/2022 (Omicron, XBB.1.5), hCoV-19/USA/WI-UW-14947/2023 (Omicron, XBB.1.5), hCoV-19/Japan/UT-NCD1288-2N/2022 (Omicron, BA.2), hCoV-19/USA/NY-MSHSP-PV56475/2022 (Omicron, BA.2.12.1), hCoV-19/Japan/UT-OM006/2022 (Omicron, BA.2.3.20), hCoV-19/Japan/TY41-768/2022 (Omicron, BS.1), hCoV-19/Japan/TY41-716/2022 (Omicron, BA.2.75), hCoV-19/USA/WI-UW-13867/2022 (Omicron, BN.1), hCoV-19/Japan/UT-OM039/2022 (Omicron, CJ.1), hCoV-19/Japan/UT-OM012/2022 (Omicron, CH.1.1), SARS-CoV-2/human/USA/WI-UW-12767/2022 (Omicron, BA.4.6), SARS-CoV-2/human/USA/COR-22–063113/2022 (Omicron, BA.5), hCoV-19/Japan/TY41-702/2022 (Omicron, BE.1), hCoV-19/Japan/UT-OM040/2022 (Omicron, BE.1.1), hCoV-19/Japan/TY41-796/2022 (Omicron, BQ.1.1), hCoV-19/Japan/UT-OM128/2023 (Omicron, CK.1.1), hCoV-19/Japan/TY41-795/2022 (Omicron, XBB.1), hCoV-19/USA/NY-MSHSPSP-PV73997/2022 (Omicron, XBB.1), and hCoV-19/Japan/IC-16225/2023 (TY41-951; Omicron, XBB.1.9.1). The following SARS-CoV-2 viruses were isolated using Vero E6-TMPRSS2-T2A-ACE2 cells at 37°C and used without further amplification: hCoV-19/Japan/UT-NC1350-1N/2023 (Omicron, XBB.1.5), hCoV-19/Japan/UT-NC1351-1N/2023 (Omicron, XBB.1.5), hCoV-19/Japan/UT-BB7644-1N/2023 (Omicron, XBB.1.5), and hCoV-19/Japan/UT-NC1367-1N/2023 (Omicron, XBB.1.5). All experiments with SARS-CoV-2 were performed in enhanced biosafety level 3 containment laboratories at the University of Tokyo, which are approved for such use by the Ministry of Agriculture, Forestry, and Fisheries, Japan.

### Growth kinetics in cultured cells

VeroE6/TMPRSS2 cells grown on 24-well plates in triplicate were infected with the indicated virus at a multiplicity of infection (MOI) of 0.001. The inoculum was removed after 60 min of incubation at 37°C, 32°C, or 30°C, and the cells were further incubated at 37°C, 32°C, or 30°C. Cell culture supernatants were collected at the indicated timepoints. Virus titers were determined by performing a plaque assay with VeroE6/TMPRSS2 cells.

### Deep sequencing analysis

Viral RNA was extracted by using a QIAamp Viral RNA Mini Kit (QIAGEN) and RNase-free DNase Set (Qiagen). The whole genome of SARS-CoV-2 was amplified by using a modified ARTIC network protocol in which some primers were replaced or added (39) . Briefly, viral cDNA was synthesized from the extracted RNA by using a LunarScript RT SuperMix Kit (New England BioLabs). The DNA was amplified by performing a multiplexed PCR in two pools using the ARTIC-N6 primers and the Q5 High-Fidelity DNA polymerase or Q5 Hot Start DNA polymerase (New England BioLabs). DNA libraries for Illumina NGS were prepared from pooled amplicons by using a QIAseq FX DNA Library Kit (QIAGEN) and were then analyzed by using the iSeq 100 System (Illumina). To determine the virus sequences, the reads were assembled by CLC Genomics Workbench (version 23, Qiagen) with the Wuhan/Hu-1/2019 sequence (GenBank accession no. MN908947) as a reference.

### BAC construction

Six overlapping DNA fragments spanning the whole SARS-CoV-2 genome were amplified by PCR using PrimeSTAR GXL DNA Polymerase (TaKaRa). Mutations were introduced into fragments by using primer-guided mutagenesis. The six fragments and the linearized pBeloBAC11 vector were assembled by using NEBuilder HiFi DNA Assembly Master Mix (NEB), resulting in infectious cDNA clones under the control of a cytomegalovirus promoter. ET SSB (NEB) was added to the reaction mix to improve the efficiency and accuracy of the assembly reaction (40). The constructed BACs were introduced into DH10B *Escherichia coli* (NEB) by electroporation. The *E. coli* was amplified at 37°C, and BACs were extracted by using NucleoBond Xtra Maxi (TaKaRa). The sequences of all constructs were confirmed by Sanger sequencing.

### Recombinant SARS-CoV-2 generation

To recover recombinant SARS-CoV-2, BACs encoding the full-length SARS-CoV-2 genome were transfected into HEK293T cells by using TransIT-293 (TaKaRa) according to the manufacturer’s protocol. At 3 days post-transfection, the supernatant containing viruses was collected and inoculated onto VeroE6/TMPRSS2 at 37°C to prepare the virus stock. The virus titers of the stock viruses were determined by using plaque assays in VeroE6/TMPRSS2. The stock viruses were deep sequenced to confirm that there were no unintended amino acid substitutions with a frequency greater than 10% in any of the virus stocks.

### Subgenomic viral RNA qRT-PCR

VeroE6/TMPRSS2 cells grown on 24-well plates in triplicate were infected with the indicated virus at an MOI of 0.5. The inoculum was removed after 60 min of incubation at 37 or 30°C, and the cells were further incubated at 37°C or 30°C. Total RNA was extracted from infected cells at the indicated timepoints by using RNeasy Mini kit (Qiagen) and RNase-free DNase Set (Qiagen). The extracted RNA was reverse-transcribed into cDNA and amplified by QuantiTect Probe RT-PCR kit (Qiagen). Real-time PCR (qPCR) was performed and analyzed on a Light Cycler96 System (Roche). The cycle conditions of qPCR were 50°C for 30 min, 95°C for 15 min, followed by 45 cycles of 95°C for 15 s, and 60°C for 60 s. The expression of E-sgRNA was analyzed by using the 2^–ΔΔCT^ method (41) and normalized to the expression of GAPDH mRNA (42). The primer and probe sequences were as follows: E-sgRNA-F: CGATCTCTTGTAGATCTGTTCTC, E-sgRNA-R: ATATTGCAGCAGTACGCACACA, E-sgRNA-probe: FAM-ACACTAGCCATCCTTACTGCGCTTCG-BHQ1 (43), GAPDH-F: GAAGGTGAAGGTCGGAGTC, GAPDH-R: GAAGATGGTGATGGGGCTTC, and GAPDH-probe: FAM-CAAGCTTCCCGTTCTCAGCC-BHQ1 (44).

### Replicon assay

The replicative cDNA of SARS-CoV-2, which lacks the structural S, M, and E genes and contains the nanoluciferase gene between the replicase and N genes with a transcription regulatory sequence of the M gene, was reported previously (15). The replicative cDNA of hCoV-19/USA/MD-HP40900-PIDYSWHNUB/2022 (HP40900) was assembled into pBeloBAC11 as described above. To introduce amino acid mutations, the BAC encoding the replicative cDNA of HP40900 was digested with PacI/MluI-HF or MluI-HF/BstbI and assembled with DNA fragments containing the intended mutations by using NEBuilder HiFi DNA Assembly Master Mix (NEB). For the replicon assay, HEK293T cells were seeded onto 24-well plates at 1.5 × 10^5^ cells/well and cultured at 37°C overnight. The BACs encoding the replicative cDNA and pGL3-control vector were transfected into the HEK293T cells using TransIT-LT1 (TaKaRa) according to the manufacturer’s instructions. pGL3 Control Vector, a gift from Debrya Groskreutz (Addgene plasmid #212937; https://www.addgene.org/212937/; RRID: Addgene_212937), expresses firefly luciferase and served as an internal control. After incubation for 72 h at 37°C, 32°C, or 30°C, the transfected cells were lysed with Passive Lysis buffer (Promega). The nanoluciferase activity and the firefly luciferase activity were measured by using the Nano-Glo luciferase assay system (Promega) and Luciferase Assay System (Promega), respectively. Polymerase activity was calculated by normalizing the nanoluciferase activity to the firefly luciferase activity. The polymerase activity of HP40900 was set to 1.

### Animal experiments

Virus inoculations were performed under anesthesia, and all efforts were made to minimize animal suffering. *In vivo* studies were not blinded, and animals were randomly assigned to infection groups. No sample-size calculations were performed to power each study. Instead, sample sizes were determined based on prior *in vivo* virus challenge experiments.

### Virus transmission in hamsters

For the airborne transmission study between hamsters, 6-week-old male hamsters were intranasally inoculated with 10^4^ PFU (in 30 µL) of the indicated virus while under isoflurane anesthesia. Infected hamsters were housed in wire cages inside an isolator unit. Twenty-four hours later, naive hamsters were placed on the other side of the cage. A double-layered wire mesh separated the hamsters by 5 cm to prevent direct contact. The infected hamsters were positioned in the front of the isolator unit, which provided unidirectional airflow. Tissue samples were collected 4 days after infection for the infected hamsters or 3 days after initial contact for the exposed hamsters. The virus titers in the nasal turbinates and lungs were determined by the use of plaque assays on VeroE6/TMPRSS2 cells.

### Statistical analysis

GraphPad Prism software was used to analyze all data. Statistical analysis was conducted by use of a *t*-test and one- or two-way analysis of variance (ANOVA) with multiple comparison post-hoc corrections. Differences among groups were considered significant for *P* values < 0.05.

## Data Availability

All data supporting the findings of this study are available within the paper and from the corresponding author upon request. There are no restrictions to obtaining access to the primary data.

## SUPPLEMENTAL MATERIAL

Supplemental tables jvi.02217-24-s0001.xlsx Tables S1 to S4.

ASM does not own the copyrights to Supplemental Material that may be linked to, or accessed through, an article. The authors have granted ASM a non-exclusive, world-wide license to publish the Supplemental Material files. Please contact the corresponding author directly for reuse.
